# Radioactive Iodine Treatment for Thyroid Cancer Patients Increases the Risk of Long-Term Gastrointestinal Disorders: A Nationwide Population-Based Cohort Analysis

**DOI:** 10.3390/cancers14102505

**Published:** 2022-05-19

**Authors:** Yueh Lee, Chi-Hsiang Chung, Li-Fan Lin, Chuang-Hsin Chiu, Yi-Feng Chen, Chao-Feng Chang, Cheng-Yi Cheng, Wu-Chien Chien

**Affiliations:** 1 Department of Nuclear Medicine, Tri-Service General Hospital, National Defense Medical Center, Taipei City 11490, Taiwan; 497010440@mail.ndmctsgh.edu.tw (Y.L.); fanlin@mail.ndmctsgh.edu.tw (L.-F.L.); treasure@mail.ndmctsgh.edu.tw (C.-H.C.); ychen8@mail.ndmctsgh.edu.tw (Y.-F.C.); 2 School of Public Health, National Defense Medical Center, Taipei City 11490, Taiwan; g694810042@mail.ndmctsgh.edu.tw; 3 Department of Medical Research, Tri-Service General Hospital, National Defense Medical Center, Taipei City 11490, Taiwan; 4 Taiwanese Injury Prevention and Safety Promotion Association, Taipei City 11490, Taiwan; 5 Division of Gastroenterology, Department of Internal Medicine, Tri-Service General Hospital, National Defense Medical Center, Taipei City 11490, Taiwan; taiwanvincent777@mail.ndmctsgh.edu.tw; 6 Graduate Institute of Life Sciences, National Defense Medical Center, Taipei City 11490, Taiwan

**Keywords:** radioactive iodine, thyroid cancer, comorbidity, gastrointestinal disorder, duodenal ulcer, gastric ulcer, chronic gastritis, secondary neoplasm of the stomach, radiation dose

## Abstract

**Simple Summary:**

The standard treatment for well-differentiated thyroid cancer is thyroidectomy followed by radioactive iodine (RAI) treatment or active surveillance. Despite adequate documentation of acute gastrointestinal adverse effects after RAI treatment, whether the gastrointestinal exposure causes long-term comorbidity or not remained unclear. We conducted a nationwide, population-based retrospective cohort study using the data from the Taiwan National Health Insurance Research Database (NHIRD) to clarify the association between long-term gastrointestinal disorders (including ulcers, atrophic gastritis, and secondary stomach malignancy) and RAI treatment in thyroid cancer patients. We found that patients with RAI treatment were at a significantly higher risk of developing gastric and duodenal ulcers than those without. In addition, a higher cumulative dose is associated with higher risk. Therefore, follow-ups at gastrointestinal clinics might be of great importance for patients presenting with chronic gastrointestinal discomforts, after receiving a single RAI dose of more than 1.11 GBq, and undergoing repeated treatment due to recurrent or residual thyroid cancer.

**Abstract:**

(1) Background: The study aimed to investigate the association between radioactive iodine (RAI) treatment and long-term gastrointestinal disorders including ulcers, atrophic gastritis, and secondary malignant neoplasm of the stomach in patients with thyroid cancer. (2) Methods: The data of the study were extracted from the National Health Insurance Database (NHIRD) of Taiwan between 2000 to 2015. Patients of ages older than 20 with thyroid cancer after thyroidectomy were included and divided into groups with RAI (study cohort) and without RAI (comparison cohort). Multivariate Cox proportional hazards regression analysis and the Kaplan–Meier method were used for statistical analysis. (3) Results: A total of 7250 (with RAI: 5800, without RAI: 1450) patients were included. The Kaplan-Meier analysis revealed a significantly higher cumulative risk for overall gastrointestinal disorders in the group with RAI (log-rank *p* = 0.034). The risk for gastrointestinal disorders was higher when receiving a cumulative RAI dose higher than 1.11 GBq in the Cox regression analysis. In the subgroup analysis, the risks of gastric and duodenal ulcers are significantly higher in the group with RAI treatment. (4) Conclusions: This study revealed that RAI was associated with an increased risk for long-term gastrointestinal disorders, specifically gastric and duodenal ulcers, in thyroid cancer, especially when the cumulative dose exceeds 1.11 GBq.

## 1. Introduction

Thyroid cancer is the most prevalent endocrine malignancy and has also become the ninth most common cancer worldwide, accounting for more than 586,000 cases in 2020 [1], with a gradual increasing incidence in both genders [2,3]. According to the 2015 American Thyroid Association (ATA) Management Guidelines, the standard treatment for well differentiated thyroid cancer, which mainly comprised papillary and follicular subtypes and accounts for most thyroid cancer cases [4,5], is surgical treatment and subsequent indicated radioactive iodine (RAI) treatment or active surveillance [6].

Since well differentiated thyroid cancer bears a relatively good prognosis among all malignancies, with a 5-year overall survival of more than 90% [1,2,5,7], the understanding of long-term comorbidities following cancer treatment is important for improving the patients’ life quality.

The potential adverse effects following RAI treatment consist of acute complications including but not limited to gastrointestinal symptoms and thyroiditis, as well as late side effects, such as xerostomia, xeropthalmia, nasolacrimal duct obstruction, bone marrow suppression, gonadal damage, metachronous primary malignancy, dental caries, alopecia, lung fibrosis, and gene mutation [8,9,10]. Among them, gastrointestinal symptoms, such as nausea and vomiting occur in about 67% of the patients and usually subside rapidly within 2 days after initiation [11].

However, whether gastrointestinal exposure to the radiation of RAI treatment causes long-term comorbidity or not remained unclear to our knowledge. To clarify the association between long-term gastrointestinal disorders (including ulcers, atrophic gastritis, and secondary malignant neoplasms of the stomach) and RAI treatment in thyroid cancer patients, we conducted this nationwide, retrospective cohort study using data from the Taiwan National Health Insurance Research Database (NHIRD).

## 2. Materials and Methods

### 2.1. Data Sources

The Taiwan NHIRD is a secondary database (accessed on 23 September 2019) containing comprehensive anonymous information regarding the demographic details, clinical visits, examinations, diagnoses, medications, and other surgical interventions derived from the medical records of the National Health Insurance (NHI) program, which was implemented in 1995 and enrolled over 99.5% of the 23 million population nationwide in Taiwan. The data of this study were extracted from the Longitudinal Health Insurance Database (LHID), which is a subset that randomly sampled 1 million enrollees from the Taiwan NHIRD.

### 2.2. Ethics Considerations

This study was approved after serial review by the Institutional Review Board of Tri-Service General Hospital (approval number TSGHIRB No. B-110-58) and adhered to the tenets of the Declaration of Helsinki. The requirement for informed consent was exempted due to the fully anonymized data source.

### 2.3. Study Design, Subjects, and Outcome

We extracted the data from the LHID during the period from 1 January 2000 to 31 December 2015, using the International Classification of Disease, Ninth Revision, Clinical Modification (ICD-9-CM) to identify the study population. Patients aged 20 years or older with thyroid cancer (ICD-9-CM 193) who underwent total thyroidectomy (ICD-9-CM 06.4) and post-treatment thyroid hormone supplement with levothyroxine were included. The included patients were divided into two groups: the with RAI (ICD-9-CM code 92.29) group as the study cohort, and the without RAI group as the comparison cohort. To reduce selection bias, the study cohort was selected according to a four-fold propensity score matching the comparison cohort by gender, age, and index date [12].

The study outcome was the diagnosis of gastrointestinal disorders including gastric ulcers (ICD-9-CM 531), duodenal ulcers (ICD-9-CM 532), peptic ulcers (ICD-9-CM 533), gastrojejunal ulcers, malignant neoplasm of the stomach (ICD-9-CM 151) and atrophic gastritis (ICD-9-CM 535.1). All the patients were followed from the index date until the occurrence of the outcome, the date of withdrawal from the insurance system, or the end of 2015. The index date was defined as the time when thyroid cancer was diagnosed, and RAI treatment was given. Patients with thyroid cancer before 1 January 2000, those who were diagnosed with the listed gastrointestinal disorders or other cancers (ICD-9-CM 140–208) before the index date, loss of tracking, age less than 20, and an unknown gender were excluded from this study.

The association between the different cumulative doses of RAI, which were subgrouped into 0.037–1.11 GBq (1–30 mCi), >1.11–3.7 GBq (>30–100 mCi), and >3.7 GBq (>100 mCi), and the risk of gastrointestinal disorder were also analyzed.

### 2.4. Comorbidities and Methods of Thyroid-Stimulating Hormone (TSH) Stimulation

We assessed the following comorbidities: *Helicobacter pylori* infection (ICD-9-CM 041.86), obesity (ICD-9-CM 278), alcoholism (ICD-9-CM 303, 305, V11.3), autoimmune disorders including chronic lymphocytic thyroiditis (ICD-9-CM 245.2) and celiac disease (ICD-9-CM 579.0), as well as Revised Charlson Comorbidity Index (CCI_R, excluding malignancies and peptic ulcer disease) [13]. The ICD-9-CM codes used in this study were listed in Table S1. We also evaluated the methods of TSH stimulation for the preparation of RAI treatment either with recombinant human TSH (rhTSH), such as Thyrotropin alpha (Trade name: Thyrogen) used in Taiwan or without.

### 2.5. Statistical Analysis

Categorical data were analyzed using the Chi-square test, while continuous variables were analyzed with the two-sample *t*-test and shown as means ± standard deviations (SD). Multivariate Cox proportional hazards regression analysis was applied to calculate the hazard ratios (HR) and 95% confidence intervals (CI) for the association of the variables (including gender, age, and comorbidities) with the development of gastrointestinal disorders. The difference in the risk of outcome between the two cohorts was determined by the Kaplan-Meier method and compared with a log-rank test. All the statistical analyses were performed using SPSS 22.0 software (SPSS Inc., Armonk, NY, USA, IBM Corp.), with statistical significance defined as *p* < 0.05.

## 3. Results

### 3.1. Characteristics of the Study and Control Cohorts

**Figure 1:**
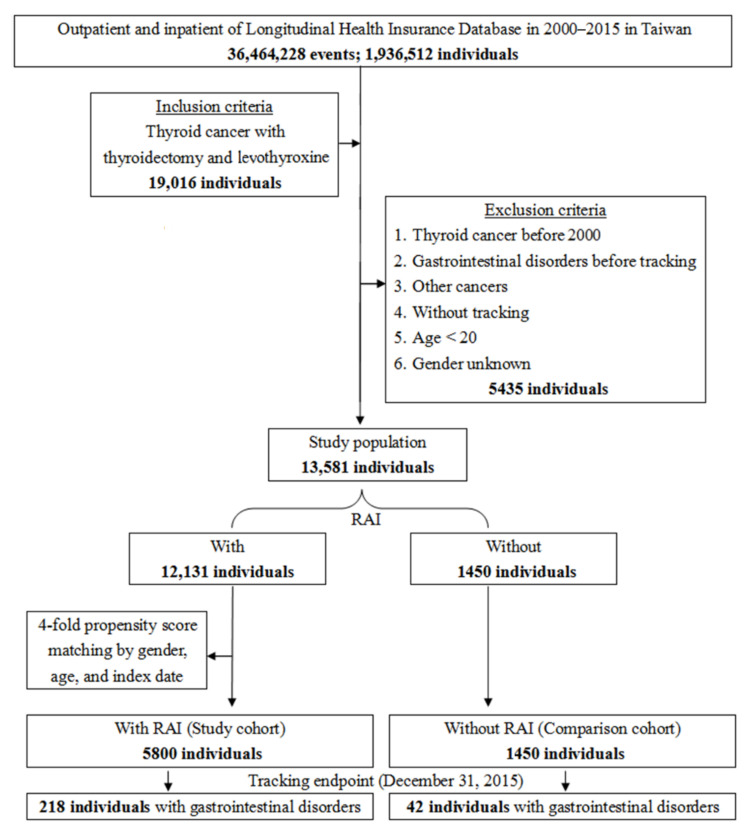
Flowchart of patient selection in this study.

A total of 19,016 patients with thyroid cancer receiving total thyroidectomy and posttreatment thyroid hormone supplement with levothyroxine were identified from the LHID database of 1,936,512 individuals from 2000 to 2015 in Taiwan. After excluding 5435 subjects according to the listed criteria, the remaining 13,581 patients were divided into two groups comprising 12,131 patients with RAI, and 1450 patients without RAI treatment (the comparison cohort). Subsequently, a fourfold group of 5800 subjects (the study cohort) from the 12,131 patients with RAI was randomly selected and matched with the comparison cohort by gender, age, and index date (Figure 1). There was no significant difference in the baseline characteristics between the two groups (Table 1).

### 3.2. Cumulative Risk of Gastrointestinal Disorders

**Figure 2:**
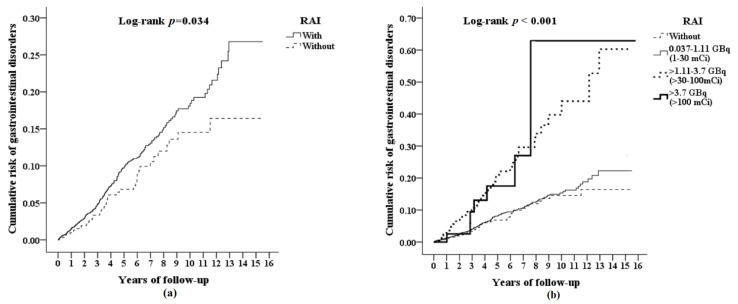
Kaplan-Meier plots with log-rank test for the cumulative risk of gastrointestinal disorders among thyroid cancer aged 20 and over stratified by (**a**) radioactive iodine (RAI); and (**b**) cumulative RAI dose.

In the Kaplan-Meier analysis, the cumulative risk of developing gastrointestinal disorders was significantly higher for patients with RAI treatment versus patients without (Log-rank *p* = 0.034, Figure 2a). When stratified by the different cumulative RAI doses, the cumulative risk was significantly higher for patients with higher RAI doses (Log-rank *p* < 0.001, Figure 2b), The mean time to develop gastrointestinal disorder was 3.82 ± 3.01 years in patients with RAI and 3.81 ± 2.78 years in patients without RAI (Table S2).

### 3.3. Risk Factors of Gastrointestinal Disorders

After adjustment for confounding factors, Cox regression multivariate analysis revealed a significantly higher hazard of developing gastrointestinal disorders in patients with RAI (adjusted HR = 1.314, 95% CI = 1.040–1.842, *p* = 0.029, Table 2).

Meanwhile, among the evaluated variables, male gender, age elder than 40 years, underlying *Helicobacter pylori* infection and higher CCI_R score were associated with a significantly higher hazard of developing gastrointestinal disorders (Table 2 and Table S3).

### 3.4. Cumulative RAI Dose and the Risk of Gastrointestinal Disorders

As shown in Table 3, the risk of developing a gastrointestinal disorder was significantly higher in patients who had undergone RAI treatment with a cumulative dose of >30–100 mCi (HR = 2.183, 95% CI = 1.497–3.578, *p* = 0.002) and greater than 100 mCi (HR = 2.209, 95% CI = 1.556–4.282, *p* = 0.001).

### 3.5. Risks of Subgroups of Gastrointestinal Disorders

Stratified subgroup analyses using multivariate Cox regression were also conducted with detailed results demonstrated in Table 4. The cohort with RAI was at significantly higher risk compared to the cohort without RAI for developing total ulcers (HR = 1.372, 95% CI = 1.075–1.927, *p* = 0.021), including its subcomponent gastric ulcers (HR = 1.456, 95% CI = 1.066–3.279, *p* = 0.032) and duodenal ulcers (HR = 3.983, 95% CI = 1.192–16.738, *p* = 0.001) among all the listed outcome subgroups. No significant risk difference was noted between the study and comparison cohorts regarding the development of malignant neoplasm of the stomach or atrophic gastritis.

## 4. Discussion

This is a nationwide population-based study with a follow-up time of 15 years using data derived from the NHIRD of Taiwan and is the first of its kind, to our knowledge, to evaluate the possible association between long-term gastrointestinal disorders and RAI treatment in thyroid cancer patients after total thyroidectomy.

We discovered that RAI treatment was associated with a statistically significant higher risk of developing long-term gastrointestinal disorders, specifically gastric and duodenal ulcers among the listed outcome subgroups.

Ionizing radiation damages the cell by direct disrupting the DNA structure or by indirect reaction via producing free radicals [14]. The deleterious altering of genetic materials might be the fundamental mechanism in all adverse effects following radiation therapy.

Orally administered RAI is absorbed via the sodium/iodine symporter (NIS) located on the surface of the microvilli or brush border in all three segments (duodenum, jejunum and ileum) of the small intestine and secreted from the bloodstream via the NIS on the basal surface of the stomach mucosa back into the gastrointestinal tract [15,16,17,18,19]. According to the dosimetry studies conducted by Johansson et al. and Brill et al., the stomach along with the bladder and salivary glands absorb the highest radiation dose among the extrathyroid organs. Retention of orally administered RAI into the gastrointestinal tract and secretion of absorbed RAI from gastric mucosa are the sources of gastrointestinal radiation exposure following RAI treatment [20,21].

Both early and late gastrointestinal toxicities following external beam radiation therapy (EBRT) have been demonstrated in the literature. Reduced gastric acid, necrosis of chief and parietal cells, mucosal thinning, inflammatory infiltration, and acute ulceration following radiation therapy are considered to be the pathogenesis [22,23]. The related acute clinical manifestations post EBRT include nausea, vomiting, dyspepsia, and abdominal pain that might start within one day after initiation and usually subsides within two weeks after the completion of radiation therapy [24,25]. The acute symptoms post-EBRT present relatively longer duration in comparison with those induced by RAI [11]. Chronic complications after EBRT include gastric ulcers, duodenal ulcers, severe gastritis, and bowel obstruction according to the study conducted by Cosset et al. on patients with Hodgkin’s disease [26].

Acute and late gastrointestinal ulceration might develop after the completion of EBRT [27]. Regarding the late ulceration of the stomach, Coia et al. suggested a usual onset of more than 5 months after EBRT [22]. Not only EBRT but also internal radiation with radionuclides aside from RAI might also cause chronic gastric ulceration, as in the case reported by Blesl et al. after selective internal radiation therapy (SIRT) with Yttrium-90 (Y-90) microspheres for cholangiocarcinoma [28]. In contrast, except for two animal studies that focused on the pharmacological protective effects of vitamin E or lycopene from gastrointestinal effects (including ulcers) after administrating high dose RAI [29,30], neither acute nor chronic gastric or duodenal ulceration was reported in humans under clinical settings after RAI treatment to our knowledge. However, the results of our study showed that thyroid cancer patients with RAI treatment were at significantly higher risk of developing gastric and duodenal ulcers than patients in the long term, and the mean time to develop a gastrointestinal disorder was 3.82 months after RAI administration.

The risk of late gastrointestinal effects appears to be associated with radiation dose. Emami et al. demonstrated that the TD5/5 (the dose at which 5 percent of patients develop complications at five years) was 50 and 40 Gy for the stomach and small intestine, respectively, when the entire organ was irradiated by EBRT [31]. The study conducted by Brick et al. also showed that the rates of gastric ulceration and perforation were higher in patients receiving RT of doses larger than 50 Gy [32]. These findings echo the results of our study that a higher dose of RAI (to more than 1.11 GBq) is associated with a higher risk of gastrointestinal comorbidity. As compared with a complete course of EBRT, the radiation dose to the stomach is usually lower in RAI. According to the model proposed by Johansson et al., the absorbed dose of the stomach per unit of RAI activity administered was 1.2 mGy/MBq [20]. Therefore, the empiric RAI treatment dose of 30–200 mCi (1.11–7.4 GBq) according to the ATA guidelines [6] may result in approximately 1.3–8.9 Gy of stomach dose. The results of our study imply that a lower accumulated radiation dose might be sufficient to cause long-term mucosal damage to the stomach and duodenum.

Among the risk factors discussed in our study, *Helicobacter pylori* infection is known for causing peptic ulcers with their association first discovered by the 2005 Nobel Prize laureates Barry Marshall and Robin Warren [33]. Our statistical analysis also revealed that *Helicobacter pylori* infection is associated with significantly higher hazards of gastrointestinal disorders. Several investigations were focused on the effect of RAI on *Helicobacter pylori* infection but showed conflicting results. Three studies revealed certain antimicrobial effects [34,35,36], while one study found no therapeutic benefit [37]. In our study, no change in infectious status among the enrolled cohorts was noted between the baseline and endpoint.

Autoimmune thyroid disorders, such as chronic lymphocytic thyroiditis (also known as Hashimoto’s thyroiditis) were shown to be associated with thyroid cancers in previous literature [38]. Besides, non-thyroidal autoimmune diseases with gastrointestinal presentations, such as celiac disease were found to be more prevalent in patients with autoimmune thyroid disorders than in the normal population [39]. Some studies examined the interplay between these disease entities [40,41] despite that, the exact mechanisms remained unclear. We have included chronic lymphocytic thyroiditis and celiac disease in the listed comorbidities for data analysis. Among the enrolled 7250 patients, 115 had chronic lymphocytic thyroiditis, and 212 had celiac disease (Table 1). However, none of the patients with these two diseases developed the gastrointestinal disorders listed in our study during the study period, and the adjusted hazard ratios did not show statistical significance (Table 2). Longer follow up time and larger case numbers might be necessary in future studies to evaluate this issue.

To achieve TSH stimulation for preparation of RAI treatment, either administration of rhTSH, such as Thyrogen, or the withdrawal of thyroid hormone (THW) supplement for 2 to 4 weeks according to the ATA guidelines can be chosen [6]. According to previous investigations, the outcomes of patients receiving RAI treatments prepared by two different methods were comparable [42,43]. However, whether the short-term hypothyroidism resulting from thyroid hormone withdrawal is related to long-term gastrointestinal disorders or not is yet to be determined. We managed to evaluate this issue by adding it to our risk factor analysis. The adjusted hazard ratio of those prepared with Thyrogen versus without for developing gastrointestinal disorders did not achieve statistical significance (adjusted HR = 2.301, 95% CI = 0.341–86.010, *p* = 0.897) as shown in Table 2. Among the 7250 enrolled patients, less than 4 percent used Thyrogen, and the rest of the patients were presumed to undergo THW as shown in Table 1. However, the percentage might be underestimated since some patients who did not qualify for the National Health Insurance reimbursement for Thyrogen (e.g., comorbid medical conditions intolerant to hypothyroidism) might still choose Thyrogen stimulation at their own expense, which was not recorded by NHIRD.

RAI treatment was reported to increase the risk of certain kinds of a second primary malignancy. A population-based study published in 2016 by Teng et al. showed a higher risk for developing leukemia, lymphoma, prostate, lung, pancreas, kidney, breast, and colorectal cancers, but not gastric cancer [44], which was in line with the results of our study, that there was no risk difference regarding the development of malignant stomach neoplasms between patients with and without RAI treatment.

According to the ATA guidelines, RAI treatment is recommended for thyroid cancer patients of high or intermediate risk groups but not routinely for those of low-risk groups. Besides, the guidelines also suggest maintaining a stricter TSH suppression, which should be achieved by a higher dose of levothyroxine supplement, for those high and intermediate risk patients [6]. In patients with multiple RAI treatments prepared with THW, a longer time of hypothyroidism state was induced. To our knowledge, there is currently no solid evidence on the role of high dose thyroid hormone supplements on long-term gastrointestinal comorbidities, but hyperthyroidism related anorexia, vomiting and diarrhea were clearly documented [45,46,47]. Furthermore, the role of hypothyroidism on gastric ulcers remained unclear. In animal experiments conducted by Koyuncu et al., thyroid hormone reduced the length and depth of stress ulcers in rats when given before or at the beginning of the stress [48]. The possible confounding effect of TSH suppression and hyper-/hypothyroidism status on the development of long-term gastrointestinal disorders after RAI treatment is unknown. However, laboratory parameters including TSH, T3 and T4 levels essential for evaluation of these issues were not recorded in the NHIRD database. We managed to evaluate TSH suppression indirectly by analyzing the prescribed levothyroxine dose as a surrogate marker. A similar method had been demonstrated previously in the research conducted by Suh et al. for cardiovascular risk in thyroid cancer patients taking levothyroxine [49]. As shown in the lowest rows of Supplementary Table S3, we divided all the enrolled patients according to their average daily dose of levothyroxine into three tertiles as <33% (60 μg/day), ≥33–67% (≥60–128 μg/day) and ≥67% (≥128 μg/day). The adjusted hazard ratios of developing gastrointestinal disorders in patients with RAI are still significantly higher versus those without RAI despite different levothyroxine daily doses. However, since the factors that might interfere with the final TSH suppression results, such as levothyroxine dose adjustment, patient compliance to prescriptions, and interruption of administration (e.g., due to treatment or imaging needs) were not readily available from the NHIRD and could not be derived from average daily dose, these issues still required further research of other experimental design for confirmation.

Some limitations exist regarding our study. The first among them is that the prescribed medications were not stratified in our study. Since NSAIDs including aspirin were reported to result in a fourfold risk increase for developing ulcers [50], the lack of related dose and treatment duration of these medications might influence the results of statistical analysis. Secondly, some candidate behavioral (such as smoking habit and non-abusive alcohol consumption) and psychosocial (such as stress) risk factors of developing gastrointestinal disorders, family history, body mass index, ATA risk stratification results, thyroid cancer staging, as well as the laboratory parameters (such as thyroglobulin levels in addition to the above mentioned TSH, T3 and T4) are not readily available from the NHIRD database. Additionally, the number of subjects having comorbidities (*Helicobacter pylori* infection, obesity, alcoholism, chronic lymphocytic thyroiditis, and celiac disease) selected in our study was too low and thereby hindered meaningful statistical analysis. Thirdly, our study only focused on the Taiwan population, which may prohibit the application of study results to other countries of different ethnic compositions. Finally, our selection of gastrointestinal disorders mainly focused on the stomach and duodenum. Future studies may aim toward the association between RAI treatment and late toxicity to the esophagus and distal parts of the intestine.

Nevertheless, this study has several strengths. Firstly, the data were derived from a national database with a large sample size and long follow-up period which provided strong statistical power for relatively rare long-term gastrointestinal comorbidities in thyroid cancer patients receiving RAI. Moreover, we used four-fold propensity score matching to minimize selection bias. Finally, a multivariate Cox regression analysis was also performed to adjust the confounding factors.

## 5. Conclusions

This study revealed that thyroid cancer patients with RAI treatment are at increased risk of developing gastrointestinal disorders, specifically gastric and duodenal ulcers. In addition, cumulative RAI doses of more than 1.11 GBq are associated with significantly higher risk. Follow-ups at gastrointestinal clinics might be of great importance, especially for patients presenting long-term gastrointestinal symptoms, receiving a single RAI dose of more than 1.11 GBq, and undergoing repeated treatment due to recurrent or residual thyroid cancer.

## Figures and Tables

**Table 1:** Characteristics of the enrollees in study and control cohorts at baseline.

Variables	Total		With RAI Study Cohort		Without RAI Comparison Cohort		*p*
	*n*	%	*n*	%	*n*	%	
**Total**	7250		5800	80.00	1450	20.00	
**Gender**							0.999
Male	1505	20.76	1204	20.76	301	20.76	
Female	5745	79.24	459	79.24	1149	79.24	
**Age (years)**	47.40 ± 13.34		47.55 ± 13.22		46.79 ± 13.77		0.052
**Age groups (years)**							0.999
20–39	2190	30.21	1752	30.21	438	30.21	
40–59	3800	52.41	3040	52.41	760	52.41	
60–79	1205	16.62	964	16.62	241	16.62	
≥80	55	0.76	44	0.76	11	0.76	
***Helicobacter pylori* infection**							0.365
Without	7248	99.97	5799	99.98	1449	99.93	
With	2	0.03	1	0.02	1	0.07	
**Obesity**							0.999
Without	7245	99.93	5796	99.93	1449	99.93	
With	5	0.07	4	0.07	1	0.07	
**Alcoholism**							0.613
Without	7249	99.99	5799	99.98	1450	100.00	
With	1	0.01	1	0.02	0	0.00	
**Chronic lymphocytic thyroiditis**							0.814
Without	7135	98.41	5707	98.40	1428	98.48	
With	115	1.59	93	1.60	22	1.52	
**Celiac disease**							0.944
Without	7038	97.08	5630	97.07	1408	97.10	
With	212	2.92	170	2.93	42	2.90	
**CCI_R**	0.10 ± 0.41		0.11 ± 0.42		0.09 ± 0.37		0.119
**rhTSH (Thyrogen)**							0.255
Without	7005	96.62	5611	96.74	1394	96.14	
With	245	3.38	189	3.26	56	3.86	

CCI_R, revised Charlson comorbidity index; rhTSH, recombinant human thyroid-stimulating hormone; RAI, radioactive iodine.

**Table 2:** Risk factors analysis for gastrointestinal disorders using Cox regression.

Variables	Crude HR	95% CI	95% CI	*p*	Adjusted HR	95% CI	95% CI	*p*
**RAI**								
Without	Reference				Reference			
With	1.360	1.077	1.892	0.034 *	1.314	1.040	1.842	0.029 *
**Gender**								
Male	1.565	1.193	2.054	0.001 *	1.395	1.068	1.849	0.011 *
Female	Reference				Reference			
**Age groups (years)**								
20–39	Reference				Reference			
40–59	1.927	1.200	3.092	0.007 *	1.869	1.157	3.015	0.009 *
60–79	2.963	1.851	4.744	<0.001 *	2.493	1.532	4.058	<0.001 *
≥80	5.292	3.132	8.943	<0.001 *	4.462	2.599	7.642	<0.001 *
***Helicobacter pylori* infection**								
Without	Reference				Reference			
With	7.511	1.052	53.604	0.044 *	8.267	1.531	75.129	0.020 *
**Obesity**								
Without	Reference				Reference			
With	0.000	−	−	0.974	0.000	−	−	0.994
**Alcoholism**								
Without	Reference				Reference			
With	0.000	−	−	0.83	0.000	−	−	0.956
**Chronic lymphocytic thyroiditis**								
Without	Reference				Reference			
With	0.000	−	−	0.979	0.000	−	−	0.993
**Celiac disease**								
Without	Reference				Reference			
With	0.000	−	−	0.988	0.000	−	−	0.997
**CCI_R**	1.458	1.302	1.633	<0.001 *	1.318	1.163	1.494	<0.001 *
**rhTSH (Thyrogen)**								
Without	Reference				Reference			
With	7.065	0.789	459.780	0.832	2.301	0.341	86.010	0.897

Adjusted HR means adjusted for variables listed in the table. RAI, radioactive iodine; CCI_R, revised Charlson comorbidity index; rhTSH, recombinant human thyroid-stimulating hormone; HR, hazard ratio; CI, confidence interval; * *p* < 0.05.

**Table 3:** Factors of gastrointestinal disorder among different dose of RAI using Cox regression.

Dose of RAI	Populations	Events	PYs	Rate (per 10^5^ PYs)	Adjusted HR	95% CI	95% CI	*p*
Without	1450	42	3169.54	1325.11	Reference			
With	5800	218	12,034.78	1811.42	1.314	1.040	1.842	0.029 *
0.037–1.11 GBq (1–30 mCi)	5391	162	10,522.26	1539.59	1.150	0.357	1.656	0.813
>1.11–3.7 GBq (>30–100 mCi)	357	48	1299.18	3694.62	2.183	1.497	3.578	0.002 *
>3.7 GBq (>100 mCi)	52	8	213.34	3749.93	2.209	1.556	4.282	0.001 *

Adjusted HR means adjusted for variables listed in the table. PYs = Person-years; RAI, radioactive iodine; HR, hazard ratio; CI, confidence interval; * *p* < 0.05.

**Table 4:** Factors of gastrointestinal disorder subgroup stratified by variables listed in the table using Cox regression.

	With RAI			Without RAI			With vs. Without (*Reference*)				
Outcome Subgroup	Events	PYs	Rate (per 10^5^ PYs)	Events	PYs	Rate (per 10^5^ PYs)	Ratio	Adjusted HR	95% CI	95% CI	*p*
**Any of the listed** **gastrointestinal disorder**	218	12,034.78	1811.42	42	3169.54	1325.11	1.367	1.314	1.040	1.842	0.029 *
Ulcer	212	12,034.78	1761.56	39	3169.54	1230.46	1.432	1.372	1.075	1.927	0.021 *
Gastric ulcer	121	12,034.78	1005.42	23	3169.54	725.66	1.386	1.456	1.066	3.279	0.032 *
Duodenal ulcer	47	12,034.78	390.53	7	3169.54	220.85	1.768	3.983	1.192	16.738	0.001 *
Peptic ulcer	44	12,034.78	365.61	9	3169.54	283.95	1.288	1.299	0.482	5.411	0.243
Gastrojejunal ulcer	0	12,034.78	0.00	0	3169.54	0.00	−	−	−	−	−
**Malignant neoplasm of stomach**	5	12,034.78	41.55	3	3169.54	94.65	0.439	0.668	0.178	2.620	0.533
**Atrophic gastritis**	1	12,034.78	8.31	0	3169.54	0.00	∞	∞	−	−	0.999

Adjusted HR means adjusted for variables listed in Table 2. PYs = Person-years; RAI, radioactive iodine; HR, hazard ratio; CI, confidence interval; * *p* < 0.05.

## Data Availability

The study extracted data from the Taiwan National Health Insurance Research Database (NHIRD), https://nhird.nhri.org.tw/en/index.html, accessed on 23 September 2019. The original contributions presented in the study are included in the article/Supplementary Material, further inquiries can be directed to the corresponding author/s.

## Supplementary Materials

The following supporting information can be downloaded at: https://www.mdpi.com/article/10.3390/cancers14102505/s1, Table S1: ICD-9-CM codes used in this study for data extraction and analysis.; Table S2: Years to Develop Gastrointestinal Disorders.; Table S3. Risk factors analysis for gastrointestinal disorders stratified by variables listed in the table by using Cox regression.
