# Supplementary material for: Radioactive Iodine Treatment for Thyroid Cancer Patients Increases the Risk of Long-Term Gastrointestinal Disorders: A Nationwide Population-Based Cohort Analysis
Source: Cancers (Basel). 2022 May 19;14(10):2505. doi: 10.3390/cancers14102505 (PMC9140163; doi:10.3390/cancers14102505)
Supplement: Supplementary file 1 [file cancers-14-02505-s001.zip › cancers-1690758-supplementary.pdf]

cancers MDPI

Supplementary Material: Radioactive Iodine Treatment for Thyroid Cancer Patients Increases the Risk of Long-Term Gastrointestinal Disorders: A Nationwide Population-Based Cohort Analysis

Yueh Lee, Chi-Hsiang Chung, Li-Fan Lin, Chuang-Hsin Chiu, Yi-Feng Chen, Chao-Feng Chang, Cheng-Yi Cheng * and Wu-Chien Chien *

Table S1. ICD-9-CM codes used in this study for data extraction and analysis.

	**ICD-9-CM / NHI Code / Definition**
**Study population**	
Thyroid cancer	193
Thyroidectomy	OP06.4
**Intervention:** Radioactive iodine treatment	OP92.29, 26038B
**Excluding:** Other cancers	140—208, except for thyroid cancer
**Outcome:** Gastrointestinal disorders	Any of the listed
Ulcer	531—534
Gastric ulcer	531
Duodenal ulcer	532
Peptic ulcer	533
Gastrojejunal ulcer	534
Malignant neoplasm of stomach	151
Atrophic gastritis	535.1
**Comorbidities**	
Helicobacter pylori infection	041.86
Obesity	278
Alcoholism	303, 305.0, V11.3
Chronic lymphocytic thyroiditis	245.2
Celiac disease	579.0
**Charlson Comorbidity Index Revised (CCI_R)**	CCI removed cancer, and peptic ulcer disease

ICD-9-CM, International Classification of Diseases, Ninth Revision, Clinical Modification; NHI, national health insurance; CCI_R, revised Charlson comorbidity index

Table S2. Years to Develop Gastrointestinal Disorders.

**RAI**	**Min**	**Median**	**Max**	**Mean ± SD**
With	0.02	3.19	12.95	3.82 ± 3.01
Without	0.09	3.39	11.53	3.81 ± 2.78
Total	0.02	3.22	12.95	3.82 ± 2.97

RAI, radioactive iodine; SD, standard deviation

cancers

MDPI

Table S3. Risk factors analysis for gastrointestinal disorders stratified by variables listed in the table by using Cox regression.

RAI	With			Without			With *vs.* Without *(Reference)*				
**Stratified**	**Events**	**PYs**	**Rate (per 10^5 PYs)**	**Events**	**PYs**	**Rate (per 10^5 PYs)**	**Ratio**	**Adjusted HR**	**95% CI**	**95% CI**	***p***
**Total**	218	12,034.78	1811.42	42	3169.54	1325.11	1.367	1.314	1.040	1.842	0.029*
**Gender**											
Male	65	2379.52	2731.64	12	620.67	1933.41	1.413	1.361	1.052	2.001	0.015*
Female	153	9655.26	1584.63	30	2548.88	1176.99	1.346	1.265	0.703	1.796	0.398
**Age groups (years)**											
20–39	16	2169.31	737.56	5	684.20	730.78	1.009	1.114	0.663	1.902	0.688
40–59	77	5297.00	1453.65	18	1350.02	1333.31	1.090	1.135	0.468	2.684	0.735
60–79	90	3719.21	2419.87	14	911.05	1536.68	1.575	1.199	1.052	3.402	0.009*
≧80	35	849.27	4121.18	5	224.26	2229.51	1.848	1.742	1.197	3.241	0.001*
***Helicobacter pylori* infection**											
Without	218	12,034.66	1811.43	41	3162.27	1296.54	1.397	1.310	1.037	1.831	0.031*
With	0	0.12	0.00	1	7.27	13,761.92	0.000	0.000	-	-	0.999
**Obesity**											
Without	218	12,034.71	1811.43	42	3169.44	1325.15	1.367	1.314	1.040	1.842	0.029*
With	0	0.07	0.00	0	0.10	0.00	-	-	-	-	-
**Alcoholism**											
Without	218	12,033.40	1811.63	42	3165.01	1327.01	1.365	1.314	1.040	1.842	0.029*
With	0	1.39	0.00	0	4.53	0.00	-	-	-	-	-
**Chronic lymphocytic thyroiditis**											
Without	218	11,841.83	1840.93	42	3121.41	1345.55	1.368	1.314	1.040	1.842	0.029*
With	0	192.95	0.00	0	48.13	0.00	-	-	-	-	-
**Celiac disease**											
Without	218	11,648.89	1871.42	42	3075.67	1365.56	1.370	1.314	1.040	1.842	0.029*
With	0	352.89	0.00	0	96.87	0.00	-	-	-	-	-
**Thyrogen**											
Without	205	11,575.45	1770.99	40	3060.57	1306.95	1.355	1.308	1.035	1.824	0.031*
With	13	459.33	2830.21	2	108.97	1835.57	1.542	1.529	1.098	1.876	0.001*
**Levothyroxine dose**											
< 33% (60 μg / day)	109	6173.25	1765.68	28	2135.27	1323.97	1.347	1.282	1.016	1.797	0.037*
≧33% (60 μg /day), <67% (128 μg / day)	75	3991.22	1879.12	9	661.08	1327.61	1.380	1.361	1.078	1.909	0.011*
≧67% (128 μg / day)	34	1870.31	1817.88	5	373.19	1327.23	1.357	1.317	1.042	1.845	0.027*

Adjusted HR means adjusted for variables listed in the table. PYs = Person-years; RAI, radioactive iodine; HR, hazard ratio; CI, confidence interval; * $p < 0.05$.
